# Extracorporeal membrane oxygenation in the care of a preterm infant with COVID-19 infection: Case report

**DOI:** 10.3389/fped.2022.953122

**Published:** 2022-08-12

**Authors:** Jessica Patrick-Esteve, Christy Mumphrey, David Yu, Emily Masoumy, Jeremy Lawson, David Hebert, Brian Barkemeyer

**Affiliations:** ^1^Department of Pediatrics-Division of Neonatology, Children's Hospital New Orleans, Louisiana State University Health Sciences Center-New Orleans, New Orleans, LA, United States; ^2^Department of Surgery, Children's Hospital of New Orleans, Tulane University School of Medicine, New Orleans, LA, United States; ^3^Department of Respiratory, Children's Hospital New Orleans, New Orleans, LA, United States

**Keywords:** COVID-19, ECMO, neonate, prematurity, case report

## Abstract

Coronavirus disease 2019 (COVID-19) was first reported to the World Health Organization (WHO) in December 2019 and has since unleashed a global pandemic, with over 518 million cases as of May 10, 2022. Neonates represent a very small proportion of those patients. Among reported cases of neonates with symptomatic COVID-19 infection, the rates of hospitalization remain low. Most reported cases in infants and neonates are community acquired with mild symptoms, most commonly fever, rhinorrhea and cough. Very few require intensive care or invasive support for acute infection. We present a case of a 2-month-old former 26-week gestation infant with a birthweight of 915 grams and diagnoses of mild bronchopulmonary dysplasia and a small ventricular septal defect who developed acute respiratory decompensation due to COVID-19 infection. He required veno-arterial extracorporeal membrane oxygenation support for 23 days. Complications included liver and renal dysfunction and a head ultrasound notable for lentriculostriate vasculopathy, extra-axial space enlargement and patchy periventricular echogenicity. The patient was successfully decannulated to conventional mechanical ventilation with subsequent extubation to non-invasive respiratory support. He was discharged home at 6 months of age with supplemental oxygen *via* nasal cannula and gastrostomy tube feedings. He continues to receive outpatient developmental follow-up. To our knowledge, this is the first case report of a preterm infant during their initial hospitalization to survive ECMO for COVID-19.

## Background

Coronavirus disease 2019 (COVID-19) was first reported to the WHO in December 2019 and has since unleashed a global pandemic, with over 518 million cases reported by May 10, 2022 (1). Neonates represent a very small proportion of those patients, with few requiring invasive support for acute infection (2, 3). Among reported cases of neonates with symptomatic COVID-19 infection, the rates of hospitalization remain low (4). Most reported cases in infants and neonates are community acquired with mild symptoms, most commonly fever, rhinorrhea, and cough (4). Very few require intensive care, with one comprehensive study reporting 3% of pediatric patients, ages 1 day to 16 years. (5). We present a case of a 2-month-old former 26-week gestation neonate who required veno-arterial extracorporeal membrane oxygenation (ECMO) support for acute respiratory decompensation due to COVID-19 infection. To our knowledge, this is the first case report of a preterm infant during their initial hospitalization to survive ECMO for COVID-19.

## Case

Our patient is a former 26-week gestation premature male infant with a birthweight of 915 grams and diagnoses of bronchopulmonary dysplasia and a small ventricular septal defect. He had a protracted NICU course notable for respiratory distress syndrome requiring surfactant therapy, intubation with mechanical ventilation for 6 days, and non-invasive positive pressure with through day of life (DOL) 52, and continued to require supplemental oxygen through DOL 68 and 36 weeks post-menstrual age, classifying his BPD as mild according to the current NICHD guidelines. Then, on DOL 82, he underwent an elective laparoscopic bilateral inguinal and umbilical hernia repair and circumcision. As per hospital policy, he had a negative COVID-19 screening test 2 days prior to this surgery. At this time, the patient weighed 2560 grams and had a stable supplemental oxygen requirement of 0.5 liters per minute FiO2 0.40 *via* nasal cannula. His parents visited regularly and were present on the day of surgery. The evening of his surgery, his mother began feeling ill and subsequently tested positive for COVID-19. The immediate post-operative course was uneventful.

**Figure 1:**
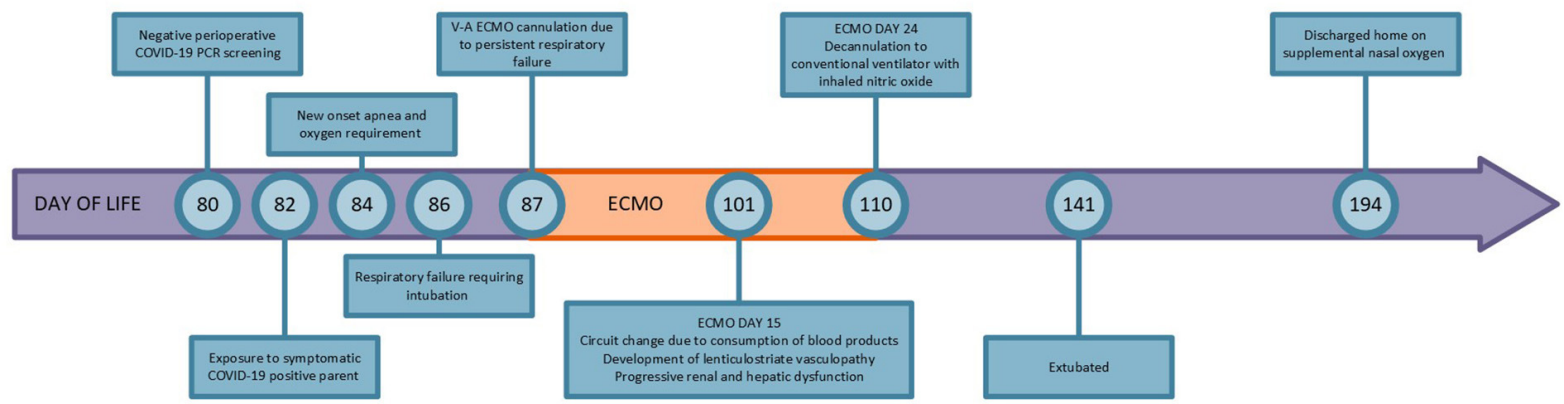
Timeline.

On postoperative day 2 (DOL 84), the patient started with new onset severe apneic episodes requiring increasing respiratory support. An evaluation for infection was performed, including a complete blood count, blood and urine cultures and a comprehensive respiratory PCR panel that included COVID-19 testing. Due to worsening clinical status, he was started on broad spectrum antibiotics and given a blood transfusion for anemia. The comprehensive respiratory PCR panel resulted positive for COVID-19, while tests for other respiratory viral pathogens and bacterial cultures were negative. On DOL 86, 2 days after the diagnosis of COVID-19, the patient had a remarkably abrupt decompensation in his respiratory status resulting in hypoxic and hypercarbic respiratory failure. Chest radiograph revealed rapidly developing bilateral dense infiltrates. Aggressive medical management including conventional ventilation, high frequency oscillatory ventilation and inhaled nitric oxide (iNO) therapy failed. Due to continued hypoxia, hypercarbia and severe acidosis, and after consultation with pediatric surgery, he was placed on veno-arterial extracorporeal membrane oxygenation (ECMO) on DOL 87, 3 days after the diagnosis of COVID-19. Cannulas needed for veno-venous ECMO were not available due to product shortage at the time of this infant's cannulation, requiring the use of veno-arterial ECMO, using an 8 French arterial and a 10 French venous cannula due to the infant's size. Once on ECMO, the patient's hypoxia, hypercarbia and acidosis resolved with the pump flow set at 120–140 milliliters/kilogram/minute, and he was anti-coagulated according to our institution's protocol using a heparin coated circuit, continuous heparin and antithrombin III infusions. Prior to ECMO cannulation his head ultrasound (HUS) showed a brain normal for age with mild prominence of the extra-axial spaces, however, approximately 12 h after cannulation a repeat HUS showed a left grade two germinal matrix hemorrhage. At this time, the anti-coagulation therapy was adjusted by decreasing the heparin infusion by 25%, according to our institutions protocol, to further reduce the anti-thrombin Xa level and minimize bleeding risk. There was no extension of the intraventricular bleed on subsequent daily HUS. Systemic hypertension necessitated the use of multiple antihypertensive agents to maintain a mean arterial blood pressure (MAP) <60 mmHg while on ECMO. Throughout the ECMO course, the patient required full ECMO support due to poor lung compliance. We were unable to offer any COVID-19 specific medicinal therapies due to insufficient data at the time that it would change the course of disease in an infant. After 15 days on ECMO, the circuit was changed due to the escalating need for multiple blood products. Around this time, serial HUS noted lentriculostriate vasculopathy, extra-axial space enlargement and patchy periventricular echogenicity. With an extended time on ECMO complicated by progressive renal and liver dysfunction, concerning HUS findings and the re-emergence of the need for multiple blood products, the patient was decannulated after 23 days on ECMO on DOL 110. Prior to decannulation, a family meeting was held to discuss treatment options, and the family requested ongoing aggressive medical management. Despite having poor lung compliance and ongoing pulmonary hypertension at the time of decannulation, he survived with short term use of inhaled nitric oxide and intravenous sildenafil. Lung compliance improved over time allowing progressive wean of ventilator support, and renal and liver function also improved to normal values. On DOL 141, he was extubated to non-invasive positive pressure ventilation with a subsequent slow wean to a low flow nasal cannula. The ventricular septal defect was smaller consistent with expected spontaneous closure. Due to the inability to feed efficiently, a gastrostomy tube was placed on DOL 182. He was discharged home with supplemental oxygen *via* nasal cannula and gastrostomy tube feeds on DOL 194, weighing 5090 grams (see Figure 1). As an outpatient, he continues to receive treatment for his pulmonary hypertension and shows neurodevelopmental progress.

## Discussion

With the onset of the COVID-19 pandemic, pediatric providers made clinical decisions based on limited information in attempt to reduce the risk of disease transmission to exposed infants and children and their providers. Initial guidance from the American Academy of Pediatrics was published online on April 2, 2020 and has been updated subsequently (6). While early disease impact was notable on pregnant women, increasing complexity of maternal care and at times leading to premature delivery, illness directly attributable to COVID-19 in infants was limited. Amongst children, multisystem inflammatory syndrome in children (MIS-C) was the greatest source of COVID-19 derived illness. With the onset of the Delta variant (7), an increasing number of pediatric cases of acute COVID-19 infection were reported, including increased severity of illness and death. Although the strain was not specifically identified, the timing of our patient's infection coincided with the Delta variant accounting for most cases in our region (8). As Delta and other novel variants un-fold in the COVID-19 pandemic, this case illustrates multiple challenges facing providers in the NICU and those caring for NICU graduates, in addition to direction for future research.

As with other respiratory viral infections, preterm infants infected with COVID-19 may present with apnea as an initial clinical finding. Early recognition of COVID-19 may expand options for therapies and reduce the likelihood of developing a severe disease course. As the treatment options for COVID-19 continue to evolve, increasing information about the efficacy of pharmacologic therapies such as monoclonal antibodies and antiviral therapies has become available for adults and older children. Complete information on the use of these products isn't available in children under age 12 (9). With consultation of pediatric infectious disease and immunology specialists, we treated with dexamethasone but did not offer any specific COVID-19 therapy to this infant. More research is needed on the use of specific COVID-19 treatments in the pediatric population, especially in younger children.

Prematurity with resultant lung disease in the form of mild bronchopulmonary dysplasia (BPD), and potentially the stress of surgery, placed our patient at increased risk for significant disease from COVID-19 (10). Other NICU patients and NICU graduates are likely at higher risk for symptomatic and severe COVID-19 infection. Efforts at disease prevention in this at-risk group should be paramount and include avoidance of sick contacts, COVID-19 vaccination of patient care providers, and when available, vaccination efforts for this susceptible population. Given the timing of the exposure and illness onset, it is likely our patient's COVID-19 infection was acquired from his mother while visiting him in the NICU, despite hospital screening procedures and universal masking for all visitors. Our patient's mother delivered prior to the third trimester where maternal vaccination may have provided some protection for the infant (11). The parents were not vaccinated which was not a requirement in our unit. However, our unit had visitors restricted to the two primary caregivers at this time with recommendations for mask wearing while in the room with their infant. In many NICUs across the country, varying degrees of visitor restrictions were enacted in attempts to limit nosocomial spread of COVID-19 to patients and health care workers (12). Balancing the necessity to protect our at-risk population while promoting infant-caretaker bonding and neurologic development continues to pose a challenge. This case highlights one side of this equilibrium, yet the impact of COVID-19 on the developmental outcomes of NICU patients may never be fully understood. Further development of methods to promote bonding and development and engaging in family centered care while limiting exposure is needed.

The developmental outcome for our patient remains uncertain, and appropriate developmental follow-up and interventions are ongoing. Our patient did develop a left grade 2 germinal matrix hemorrhage after initiation of ECMO that resolved and had subsequent findings of lenticulostriate vasculopathy. The etiology and consequence of lenticulostriate vasculopathy in this case is uncertain as it is reported to occur in 15% of preterm infants and is not independently associated with adverse neurodevelopmental outcomes (13). While COVID-19 infection is associated with a higher risk of thrombotic events in children and adults, the impact of COVID-19 infection on coagulopathy in infants is uncertain (14).

Although the literature is scarce regarding the use of ECMO support for COVID-19 pediatric and neonatal patients, the ELSO guidelines support its use if indicated, with decision to cannulate based upon “existing indications and thresholds per currently published guidelines” (15). Since the patient carried a diagnosis of Mild BPD, was in a relatively healthy state with low O2 requirements nearing discharge, with reasonable chance of recovery, we chose to place the patient on ECMO. At the time of this report, there have only been a few cases reported of neonates who were supported on ECMO for COVID-19. Both reported cases involved neonates with congenital heart disease, one pre- and the other post-op with only one of those neonates surviving to discharge (16, 17). Compared to other published case reports, our patient had a more rapid progression from diagnosis to intubation and need for ECMO support, and he also had a longer duration of ECMO (18). We suspect that the comorbidities of prematurity and BPD may have impacted the rapidity of our patient's decline and prolonged need for ECMO support, likely increasing his risk for morbidity and mortality.

To our knowledge, this is the first case report of a preterm infant during their initial hospitalization to survive ECMO for COVID-19. Our rare case serves as a reference point for neonatologists as this pandemic continues to affect our unique patient population.

## Data availability statement

The original contributions presented in the study are included in the article/supplementary material, further inquiries can be directed to the corresponding author.

## Author contributions

JP-E, CM, and EM gathered the patient data, drafted the initial manuscript, and reviewed and revised the manuscript. JL aided in drafting the initial manuscript and reviewed and revised the manuscript. BB and DY reviewed the manuscript for intellectual content and reviewed and revised the manuscript. All authors contributed to manuscript revision, read, and approved the submitted version.

## Conflict of interest

The authors declare that the research was conducted in the absence of any commercial or financial relationships that could be construed as a potential conflict of interest.

## Publisher's note

All claims expressed in this article are solely those of the authors and do not necessarily represent those of their affiliated organizations, or those of the publisher, the editors and the reviewers. Any product that may be evaluated in this article, or claim that may be made by its manufacturer, is not guaranteed or endorsed by the publisher.
